# Comparative indicators of atherogenicity, body weight, gender differences in the group of depressive and non-depressive patients with cardiovascular diseases

**DOI:** 10.1192/j.eurpsy.2021.1838

**Published:** 2021-08-13

**Authors:** N. Kornetov, O. Molodykh, A. Arzhanik

**Affiliations:** 1 Department Of Psychiatry, Addiction And Psychotherapy, Siberian States Medical Unifersity, Tomsk, Russian Federation; 2 Department Of Psychiatry, Addiction And Psychotherapy, 5th year student of the medical faculty of the Siberian State Medical Unifersity, Seversk, Russian Federation; 3 Mathematics And Computer Science, Saint Petersburg State University, Saint Petersburg, Russian Federation

**Keywords:** Depressive Disorder, Cardiovascular diseases, obesity, Dyslipidemia

## Abstract

**Introduction:**

Many studies show that obesity, dyslipidemia, and physical inactivity are closely related to depressive spectrum of symptoms (DSS), depressive disorder (DD). DD significantly reduces the patient’s quality of life and vital activity.

**Objectives:**

To study laboratory and physical parameters with DSS, DD in patients with cardiovascular diseases (CVD) to determine the number of patients requiring complex antidepressant therapy.

**Methods:**

The cross-sectional study of 127 inpatients with CVD was conducted. Depression and anxiety symptoms were evaluated using HADS, anhedonia by Snaith-Hamilton Pleasure Scale (SHAPS) and pain by visual analog scale (VAS). Acquired data was statistically processed.

**Results:**

The non-depressive patients was observed in 67 (53.0%) and 60 (47%) with DSS in CVD patients. When clinical assessing 29 (22.5%) met the criteria for major (DD), 39 (31%) for minor DD. When comparing body mass index (BMI) in patients with depression, the indicator was 31 (28.5; 33.5), in patients without depression 30 (26; 32) p <0.2828; atherogenic coefficient in patients with depression was 2.93 (2.41; 3.575), in non-depressive patients - 2.375 (2.07; 3.07) p<0.0083.

**Conclusions:**

More than 1/5 of patients with CVD need antidepressant therapy. >90% of all patients had a high BMI regardless of gender and the presence of depression. Depressive patients in 95% of cases were obese or overweight. Severe dyslipidemia in women with depression increases the risk of CVD. Conflict of interest: No.

**Disclosure:**

No significant relationships.

